# Bacterial Isolates from Bronchoalveolar Lavage in Pediatric Patients with Protracted Bacterial Bronchitis or Bronchiectasis: A Retrospective Comparative Study

**DOI:** 10.3390/jcm14217653

**Published:** 2025-10-28

**Authors:** Dafni Moriki, Maria Tsouprou, Spyridon Prountzos, Despoina Koumpagioti, Michalis Kalogiannis, Efthymia Alexopoulou, Konstantinos Douros

**Affiliations:** 1 Pediatric Allergy and Respiratory Unit, 3rd Department of Pediatrics, “Attikon” University Hospital, School of Medicine, National and Kapodistrian University of Athens, 12462 Athens, Greece; dmoriki@med.uoa.gr (D.M.); mariatsoup@gmail.com (M.T.); mixalis_kalogiannis11@hotmail.com (M.K.); 2 Department of Pediatrics, 1st Pediatric Clinic, Agia Sofia Hospital, 11527 Athens, Greece; 3 2nd Department of Radiology, “Attikon” University Hospital, School of Medicine, National and Kapodistrian University of Athens, 12462 Athens, Greece; spyttt@gmail.com (S.P.);; 4 Department of Nursing, University of West Attica, 12243 Athens, Greece; dkoumpagioti@uniwa.gr

**Keywords:** protracted bacterial bronchitis, bronchiectasis, bronchoalveolar lavage, pediatric, lower airway infection, polymicrobial infection

## Abstract

**Background:** Protracted bacterial bronchitis (PBB) and bronchiectasis share common clinical and microbiological features, but direct comparative data in children are limited. **Objectives:** To compare bronchoalveolar lavage (BAL) microbiology between pediatric PBB and bronchiectasis and identify predictors of lower airway and polymicrobial infections. **Methods:** We retrospectively reviewed children diagnosed with PBB or bronchiectasis at a tertiary center (January 2019–June 2025) who underwent both high-resolution computed tomography of the chest and bronchoscopy with BAL within a 6-month period. Multivariable logistic regression was used to identify predictors of lower airway and polymicrobial infections, adjusting for age, gender, tracheomalacia/bronchomalacia, asthma, and Bhalla score. **Results:** Among 135 children (85 with PBB, 50 with bronchiectasis), those with bronchiectasis were older (median 7.8 vs. 4.2 years, *p* < 0.001), while comorbidities showed statistically non-significant differences. The prevalence of lower airway infection was high (PBB 81.2%, bronchiectasis 74.0%; *p* = 0.330). Pathogen distribution demonstrated statistically non-significant differences between groups after adjustment, with *Haemophilus influenzae* being the most common pathogen in both groups. *Moraxella catarrhalis* was more frequent in PBB in unadjusted analysis (21.2% vs. 8.0%; *p* = 0.045), but this difference did not persist after adjustment. Polymicrobial infection occurred in 25.9% of PBB and 16.0% of bronchiectasis cases (*p* = 0.180). In regression analyses, younger age independently predicted polymicrobial infection (adjusted OR 0.81, 95% CI 0.69–0.95). **Conclusions:** BAL microbiology showed statistically non-significant differences between PBB and bronchiectasis, supporting the concept of a disease continuum. Younger children were more prone to polymicrobial infection. These findings support early targeted antibiotic therapy and close clinical surveillance to prevent progression to irreversible airway damage.

## 1. Introduction

Chronic wet cough in children is most commonly caused by protracted bacterial bronchitis (PBB) or bronchiectasis, two entities that often present overlapping clinical and microbiological findings but differ in terms of prognosis and long-term management. PBB is characterized by a persistent wet cough lasting more than four weeks, which subsides after appropriate antibiotic treatment and in the absence of alternative diagnoses [1]. In contrast, bronchiectasis represents structural damage to the airways, radiologically confirmed by high-resolution computed tomography (HRCT) showing a broncho-arterial ratio (BAR) greater than 0.80 in children [2].

Emerging evidence supports the concept of a clinical and pathophysiological spectrum between PBB and bronchiectasis, with recurrent or inadequately treated PBB potentially progressing to bronchiectasis [3,4]. Longitudinal data show that recurrent PBB (≥3 episodes/year) and isolation of *Haemophilus influenzae* from bronchoalveolar lavage (BAL) are strong independent predictors of bronchiectasis development, with the latter increasing the risk more than sevenfold [3]. These findings are consistent with the “vicious cycle” hypothesis of chronic suppurative lung disease (CSLD), in which persistent infection and neutrophil-mediated inflammation perpetuate airway damage [5]. In addition to this classical “vicious cycle” model, recent research emphasizes the evolving microbiome perspective, where airway dysbiosis and biofilm persistence play key roles in sustaining chronic infection and inflammation, further driving structural injury [6,7].

Accurate characterization of lower airway microbiology is essential for diagnosis, targeted therapy, and prognosis in both conditions. Flexible bronchoscopy with BAL remains the reference standard for pathogen detection in children, particularly when non-invasive sampling is inconclusive [8]. In both PBB and bronchiectasis, BAL most often yields *H. influenzae*, *Streptococcus pneumoniae*, and *Moraxella catarrhalis*, sometimes in polymicrobial combinations [9,10,11,12]. Although several studies have described BAL microbiology in either PBB or bronchiectasis, direct comparative data in children remain scarce [6,13]. Most prior investigations have analyzed the two conditions separately or included heterogeneous age groups, which limits conclusions about shared versus distinct microbial features. Notably, de Vries et al. [13] compared heterogeneous groups of suppurative lung diseases, while Marsh et al. [6] focused mainly on biofilm characterization without directly comparing BAL culture profiles between PBB and bronchiectasis.

Our study addresses this gap by providing a direct, parallel comparison of BAL microbiology between rigorously defined PBB and bronchiectasis cohorts, evaluated within a single tertiary referral center using standardized bronchoscopy protocols, consistent BAL culture methods, and uniform diagnostic criteria. Furthermore, we applied multivariable logistic regression to identify independent predictors of lower airway and polymicrobial infections, thereby enhancing the analytical depth and novelty of this work.

We hypothesized that the BAL microbial spectrum in PBB and bronchiectasis would overlap substantially, supporting a disease continuum.

## 2. Materials and Methods

### 2.1. Study Design and Setting

This retrospective observational study was conducted in the Pediatric Pulmonology Unit of “Attikon” University Hospital in Athens, one of the main tertiary referral centers for pediatric pulmonary diseases in Greece. All children who met the inclusion criteria during the study period (January 2019–June 2025) were enrolled consecutively to ensure comprehensive case capture and minimize sampling bias. Flexible bronchoscopy with BAL and chest HRCT were performed based on clinical indications determined by the treating physicians as part of routine diagnostic evaluation. As the study population comprised children undergoing clinically indicated bronchoscopy, a degree of selection bias cannot be entirely excluded.

### 2.2. Study Population

Children aged 0–16 years were eligible for inclusion if they met the diagnostic criteria for either PBB or bronchiectasis and had undergone both flexible bronchoscopy with BAL and HRCT of the chest within six months. Patients were excluded if one or both procedures had not been performed or if the interval between procedures exceeded six months.

The diagnosis of PBB was based on the presence of chronic wet cough lasting more than four weeks, documented improvement of symptoms after treatment with appropriate antibiotics, and exclusion of other causes [1]. Bronchiectasis was diagnosed based on compatible clinical features and confirmation with chest HRCT, defined as bronchial dilation with a BAR greater than 0.80 [2].

To ensure that all included cases represented PBB or bronchiectasis of unknown etiology, a standardized diagnostic work-up was undertaken to exclude secondary causes. Investigations in all patients included sweat chloride testing and/or *CFTR* gene mutation analysis, as well as basic immunologic screening (quantitative serum IgG, IgA, IgM, and IgG subclasses, and, when indicated, immunophenotyping and assessment of specific vaccine antibody responses). Nasal nitric oxide testing and high-speed video-microscopy analysis were performed in children who, in addition to chronic wet cough, exhibited at least one clinical characteristic suggestive of primary ciliary dyskinesia (PCD), namely, organ laterality defect, unexplained neonatal respiratory distress, early-onset year-round nasal congestion, or recurrent/persistent otitis media.

**Figure 1:**
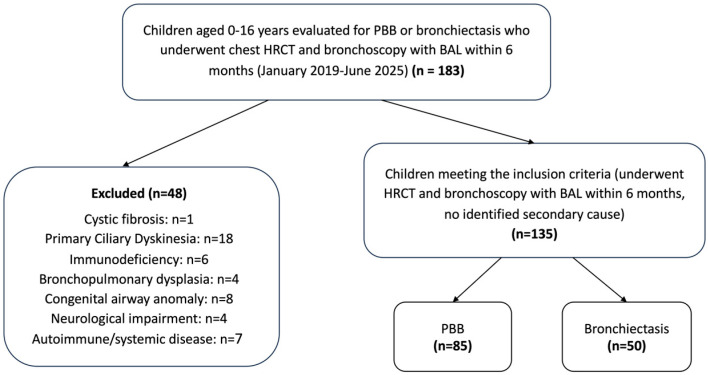
Flow diagram of patient inclusion and exclusion.

Children with confirmed diagnoses of cystic fibrosis (CF), PCD, primary or secondary immunodeficiency, bronchopulmonary dysplasia, congenital airway abnormalities (e.g., tracheoesophageal fistula), autoimmune disease, or neurological disorders affecting airway protection or clearance were excluded from the study. Children with a documented history of recurrent aspiration were also excluded, as this condition represents an alternative cause of lower-airway infection. This ensured that the final study population reflected children with PBB or bronchiectasis of idiopathic or unknown cause. A STROBE-style flow diagram (Figure 1) illustrates patient screening, exclusions by category, and the final study cohort.

### 2.3. Data Collection

Demographic data (age, gender), comorbidities (including asthma and tracheomalacia/bronchomalacia), BAL microbiology results, and Bhalla scores derived from chest HRCT scans were extracted from medical records.

Flexible bronchoscopy with BAL was performed at our center according to the European Respiratory Society (ERS) guidelines [14]. The procedures were carried out under general anesthesia, either transnasally or via a laryngeal mask airway (LMA). Measures were taken to minimize contamination of lower-airway samples by upper-airway secretions, including avoiding suctioning until the bronchoscope was below the carina and, when using an LMA, by passing the oropharynx [15,16]. All procedures were performed under sterile conditions using disposable suction catheters and sterile collection traps. At the time of bronchoscopy, none of the children had an acute illness or had received antibiotics for at least two weeks.

For BAL collection, sterile saline solution was instilled in three aliquots of 1 mL/kg (maximum 20 mL). In cases of localized disease, lavage was directed at the two most affected segments, whereas in cases of generalized disease, it was directed toward the right middle lobe and lingula. The first aliquot was used for microbiological analysis, which included quantitative cultures of aerobic bacteria performed using standard microbiological techniques. BAL samples were immediately transported at ambient temperature to the hospital’s microbiology laboratory and processed within one hour of collection to ensure sample integrity and minimize contamination risk [14,15]. Samples were cultured on standard microbiological media, including blood agar, chocolate agar, and MacConkey agar. Plates were incubated aerobically at 35–37 °C with 5% CO_2_ for 24–48 h. After incubation, colony growth was evaluated, and quantitative culture results were interpreted by a senior clinical microbiologist who was blinded to participants’ clinical diagnoses and imaging findings. Routine aerobic and microaerophilic cultures were performed. However, anaerobic cultures were not systematically included, as the study focused on the predominant pathogens associated with PBB and bronchiectasis in children. Pathogen detection was defined as the presence of ≥10^4^ colony-forming units (CFU)/mL of a single organism in BAL culture [1,3]

The diagnosis of asthma was made according to the ERS clinical practice guidelines for children aged 5–16 years [17] and the ERS statement on wheezing disorders in preschool children [18]. The diagnostic criteria included a history of recurrent episodes of wheezing, coughing, or shortness of breath, with documented improvement after administration of short-acting β_2_-agonists and/or inhaled corticosteroids. In children able to undergo spirometry, an increase of ≥12% in forced expiratory volume in one second after bronchodilator administration was considered confirmatory. In preschool children who were unable to undergo reliable pulmonary function testing, diagnosis was based on a consistent clinical pattern, documented response to asthma treatment, and exclusion of alternative causes. A pediatric pulmonologist confirmed all asthma diagnoses before study inclusion.

Tracheomalacia and/or bronchomalacia were defined as a dynamic reduction in airway lumen of more than 50% during spontaneous exhalation, as visualized on flexible bronchoscopy [19]. The diagnosis was made by a pediatric pulmonologist during real-time endoscopic evaluation, with the degree of collapse assessed in relation to the maximum diameter of the airways during inspiration.

All HRCT scans were performed using a multi-detector CT scanner at Attikon University Hospital, using a volumetric acquisition protocol. HRCT chest scans were evaluated using the Bhalla scoring system [20], which was originally developed for adult patients with CF, but was subsequently validated and adapted for the assessment of lung structural changes in children, including those with non-CF bronchiectasis [21]. This semi-quantitative system evaluates multiple radiological features, including the severity and extent of bronchiectasis, peribronchial thickening, mucus plugging, sacculations or abscesses, generations of bronchial divisions involved, and the extent of mosaic perfusion. Each component is graded on a predefined scale, and the individual scores are summed to yield a total score ranging from 0 to 25, with higher scores indicating more severe disease. The Bhalla score was applied only to children with bronchiectasis, as by definition, there were no structural abnormalities in children with PBB. All chest HRCT scans in this study were independently scored by a single pediatric radiologist (S.P.), blinded to clinical and microbiological data, to minimize bias.

### 2.4. Outcomes and Definitions

The primary outcome was the presence of a lower airway infection, defined as the isolation of any bacterial pathogen at ≥10^4^ CFU/mL in BAL fluid. A secondary outcome was the presence of polymicrobial infection, defined as the isolation of more than one bacterial species at significant levels from a single BAL sample.

### 2.5. Statistical Analysis

Continuous variables were presented using medians and interquartile ranges (IQRs) and compared using the Mann–Whitney U test. Categorical variables were presented as frequencies and percentages, and compared using the Chi-square or Fisher’s exact test, as appropriate. Normality of continuous variables was evaluated using the Shapiro–Wilk test, and non-parametric methods were applied because several variables showed non-normal distributions. Logistic regression models were used to identify predictive factors of lower airway infection and polymicrobial infection, adjusting for potential confounders. The assumption of linearity in the logit for the continuous variable (age) was examined using the Box-Tidwell approach and was found to be satisfied. Multicollinearity among covariates was assessed using variance inflation factors (VIFs), all of which were <2.0, indicating no significant collinearity. Model goodness-of-fit was assessed using the Hosmer–Lemeshow test (*p* > 0.05 for all models) and quantified using the Nagelkerke pseudo-R^2^ statistic to provide an estimate of explained variance. Missing data were <5% and were handled by complete-case analysis. Odds ratios (ORs) and 95% confidence intervals (CIs) were reported, and a two-sided *p*-value of <0.05 was considered statistically significant. To assess the potential influence of age, a sensitivity analysis was performed stratifying participants into two age groups (<5 years and ≥5 years) to examine whether the association between diagnosis and polymicrobial infection remained consistent across strata. Statistical analyses were performed using SPSS for Mac, version 30.0 (SPSS Inc., Chicago, IL, USA).

## 3. Results

### 3.1. Patient Characteristics

The flow of patient inclusion and exclusion is presented in Figure 1. Among 183 children evaluated for chronic wet cough who underwent both HRCT and bronchoscopy with BAL during the study period, 48 were excluded owing to identified underlying conditions, such as CF, PCD, primary or secondary immunodeficiency, congenital airway anomalies, bronchopulmonary dysplasia, or neurological impairment, leaving 135 children in the final analysis (85 with PBB and 50 with bronchiectasis). The proportion of boys did not differ between the groups (56.5% vs. 58.0%, *p* = 0.862). Patients with bronchiectasis were significantly older than those with PBB (median 7.8 years [IQR 5.7–12.1] vs. 4.2 years [IQR 2.2–6.1], *p* < 0.001). The prevalence of tracheomalacia and/or bronchomalacia (41.2% vs. 42.0%, *p* = 0.925) and asthma (17.6% vs. 22.0%, *p* = 0.536) did not differ significantly between the groups. The demographic and clinical characteristics of the two groups are presented in Table 1.

### 3.2. BAL Microbiology

**Figure 2:**
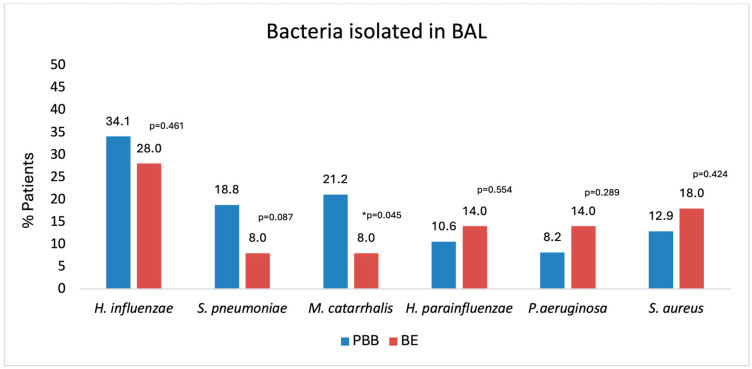
Percentage of children with protracted bacterial bronchitis (PBB, blue bars) and bronchiectasis (BE, red bars) in which specific bacterial pathogens were isolated from bronchoalveolar lavage (BAL) cultures. *H. influenzae* was the most common pathogen isolated in both groups, followed by *S. pneumoniae* and *M. catarrhalis*. * Only *M. catarrhalis* showed a statistically significant difference between the groups in the unadjusted analysis (*p* = 0.045), occurring more frequently in PBB.

Lower airway infection, defined as the isolation of any bacterial pathogen at a concentration ≥ 10^4^ CFU/mL, was detected in 81.2% of children with PBB and 74.0% of children with bronchiectasis (*p* = 0.327). The overall distribution of BAL pathogens did not differ significantly between the groups (Figure 2), with *H. influenzae* being the most frequently isolated organism in both PBB (34.1%) and bronchiectasis (28.0%).

In unadjusted analyses, the only pathogen that showed a statistically significant difference in prevalence was *M. catarrhalis*, which was more common in PBB (21.2%) compared to bronchiectasis (8.0%) (*p* = 0.045). No significant differences were observed between groups for *H. influenzae*, *S. pneumoniae*, *Haemophilus parainfluenzae*, *Pseudomonas aeruginosa*, *Staphylococcus aureus*, or other Gram-negative bacteria.

Polymicrobial infection was detected in 25.9% of children with PBB and in 16.0% of those with bronchiectasis (*p* = 0.182). In a sensitivity analysis stratified by age, the proportions of polymicrobial infection did not differ significantly between diagnostic groups in either age stratum (<5 years: 26.9% vs. 33.3%, *p* = 0.699; ≥5 years: 24.2% vs. 12.2%, *p* = 0.225), supporting the robustness of the main finding.

Overall, our findings suggest that, apart from the higher percentage of *M. catarrhalis* in PBB, the BAL microbiological profiles of the two groups are largely comparable.

### 3.3. Logistic Regression Analyses

Logistic regression analyses were performed to examine predictors of lower airway infection and polymicrobial infection (Table 2). In both unadjusted and adjusted models, the primary diagnosis (bronchiectasis vs. PBB) was not significantly associated with either outcome, suggesting that structural changes in the airways did not independently influence the risk of infection. After adjusting for age, gender, tracheomalacia/bronchomalacia, and asthma, younger age emerged as the only significant predictor, being associated with higher odds of polymicrobial infection (adjusted OR 0.81, 95% CI 0.69–0.95, *p* = 0.009). No significant associations were found for gender and comorbidities.

Diagnostic evaluation of the logistic regression models demonstrated adequate fit (Hosmer–Lemeshow *p* > 0.05 for all models) and no evidence of problematic collinearity (VIFs < 2). The assumption of linearity between age and the log odds of infection outcomes was met, supporting the validity of the regression results. Model fit indices indicated acceptable explanatory power, with Nagelkerke pseudo-R^2^ values of 0.06 for the lower airway infection model and 0.14 for the polymicrobial infection model.

To further explore this relationship, a separate logistic regression model was applied to children with bronchiectasis (*n* = 50), using the same covariates as in the previous model, with the addition of the Bhalla score. The results were consistent with those observed in the overall cohort: no variable predicted lower airway infection, whereas younger age independently predicted polymicrobial infection (OR 0.56, 95% CI 0.33–0.92, *p* = 0.023).

### 3.4. Pathogen-Specific Models

Multivariate logistic regression analyses were also performed to assess whether the odds of isolating individual pathogens differed between children with PBB and those with bronchiectasis (Table 3). After adjusting for age, gender, tracheomalacia/bronchomalacia, and asthma, there were no statistically significant differences in the likelihood of isolation of *H. influenzae*, *S. pneumoniae*, *M. catarrhalis*, *H. parainfluenzae*, *P. aeruginosa*, *S. aureus*, or other Gram-negative bacteria between the two diagnostic groups. These findings suggest that the overall lower airway microbial profile is largely similar in PBB and bronchiectasis, irrespective of structural lung changes or comorbidities.

In an exploratory analysis limited to children with bronchiectasis (*n* = 50), we examined correlations between Bhalla scores and specific bacterial pathogens using Spearman’s rank correlation. No statistically significant associations were identified between Bhalla score and the presence of *H. influenzae* (ρ = −0.05, *p* = 0.720), *S. pneumoniae* (ρ = 0.02, *p* = 0.910), *M. catarrhalis* (ρ = −0.26, *p* = 0.070), *H. parainfluenzae* (ρ = 0.12, *p* = 0.390), *P. aeruginosa* (ρ = 0.03, *p* = 0.840), *S. aureus* (ρ = −0.06, *p* = 0.690), or other Gram-negative species (ρ = 0.20, *p* = 0.160). Thus, structural disease severity, as quantified by the Bhalla score, did not correlate with isolation of specific bacterial pathogens.

## 4. Discussion

In this retrospective study of children undergoing bronchoscopy with BAL, we observed broadly similar microbiological profiles between PBB and bronchiectasis, both dominated by *H. influenzae*. This overlap reinforces the view that the two conditions lie along a shared pathophysiological spectrum rather than representing distinct entities [22]. The slightly higher occurrence of *M. catarrhalis* in PBB in unadjusted analyses may reflect age-related or transient colonization patterns rather than a disease-specific pathogen. This variation likely reflects microbial succession within the developing airway microbiota, where early-colonizing organisms such as *M. catarrhalis* are gradually replaced by other species as the airway environment and host immunity mature. Increasing mucosal immune competence, particularly enhanced IgA-mediated clearance and adaptive immune memory, may further reduce its persistence in older children [23,24].

The comparable microbial composition across groups suggests that airway infection and inflammation, rather than structural damage per se, are key drivers of symptom persistence in both conditions. The predominance of *H. influenzae*, a pathogen linked to neutrophil-mediated inflammation, biofilm formation, and recurrent infection, supports its central role in sustaining the “vicious cycle” of CSLD [25,26]. The absence of significant microbiological differences between PBB and bronchiectasis in the adjusted analyses supports the hypothesis that both conditions represent stages of the same disease spectrum [3,4]. However, given the retrospective design, causality cannot be inferred. Our findings indicate an association between the two conditions, but do not confirm direct progression from PBB to bronchiectasis. In this context, recurrent or inadequately treated episodes of PBB may contribute to airway injury and the eventual development of bronchiectasis, consistent with the “vicious cycle” model, in which persistent bacterial infection drives inflammation, tissue remodeling, and reinfection [5]. The identification of similar pathogens in both disease states indicates that early intervention in PBB, particularly when *H. influenzae* is isolated in BAL, may interrupt this cycle and prevent the structural lung damage that leads to bronchiectasis.

Notably, the Bhalla score, a marker of structural airway disease severity, did not correlate with the presence of specific pathogens, suggesting that infection burden and the extent of radiologic damage may evolve independently in pediatric bronchiectasis. Collectively, these findings emphasize the importance of timely detection and targeted treatment of bacterial infection in children with chronic wet cough to prevent airway remodeling and potential progression from PBB to bronchiectasis, in line with current guideline recommendations [9,27,28].

Our findings are further supported by longitudinal studies demonstrating that recurrent PBB and BAL isolation of *H. influenzae* are significant predictors of subsequent bronchiectasis development. In a prospective cohort study by Wurzel et al. [3], children with recurrent PBB (≥3 episodes per year) had a substantially higher likelihood of developing bronchiectasis during follow-up compared with those with isolated episodes. Consistent with this, Goyal et al. [29], in a retrospective cohort study, observed that children with persistent wet cough and ongoing bacterial infection were more likely to develop bronchiectasis, supporting the link between infection-driven inflammation and airway damage. These data place our cross-sectional findings within a longitudinal continuum, suggesting that PBB and bronchiectasis represent sequential manifestations of the same underlying pathophysiological process, where early and effective treatment of bacterial infection could prevent irreversible airway damage.

Polymicrobial infection has been well documented in children with CSLD, with reported prevalence rates ranging from 30% to 50% in those with PBB [9]. In the present study, polymicrobial infection was detected in 25.9% of children with PBB and in 16.0% of children with bronchiectasis, rates that are at the lower end of the previously reported ranges. Notably, older age was associated with a reduced likelihood of polymicrobial infection, which may reflect age-related changes in immune function, airway clearance, and microbial competition within the lower airway niche, as described in recent pediatric studies [30,31]. The clinical relevance of polymicrobial infection is underscored by accumulating evidence indicating that microbial interactions, including biofilm formation, may contribute to persistent airway inflammation and reduced response to antibiotics [7]. However, the absence of systematically collected outcome data, such as symptom resolution, recovery time, or recurrence, prevented assessment of whether pathogen burden influences clinical course. The observed inverse association between age and polymicrobial infection likely reflects the gradual maturation of host immune defenses and diversification of the airway microbiome. In younger children, incomplete development of mucosal and adaptive immunity, including lower secretory IgA levels and limited immunological memory, may allow simultaneous colonization by multiple bacterial species [32,33]. With increasing age, enhanced immune regulation and the establishment of a more stable and competitive airway microbiome may restrict the persistence of polymicrobial communities, thereby reducing the likelihood of concurrent infections [30,34,35]. Collectively, these findings support the view that the airway microbiota in children with CSLD is dynamic and evolves with age, highlighting the need for longitudinal studies to elucidate the implications of polymicrobial colonization for disease progression and treatment outcomes.

From a clinical perspective, our findings underscore the limited discriminatory value of BAL microbiology alone in distinguishing PBB from bronchiectasis. Therefore, diagnosis should continue to be based primarily on clinical course and HRCT findings. Nonetheless, BAL remains an essential tool for guiding targeted antibiotic therapy, particularly in refractory or atypical cases [8]. In this context, BAL results should be interpreted alongside clinical and imaging features as part of an integrated diagnostic algorithm. Integrating microbiological data with HRCT scoring systems (e.g., the Bhalla score), clinical history, and treatment response may improve diagnostic accuracy and enable earlier identification of children at risk of progression from PBB to bronchiectasis. Such a multidimensional approach aligns with current ERS recommendations and CHEST guidelines, which advocate a comprehensive assessment that combines clinical, radiological, and microbiological data to guide diagnosis and management [9,27,28].

Furthermore, this study contributes to the limited literature by providing a direct, parallel comparison of PBB and bronchiectasis using standardized bronchoscopy protocols, HRCT scoring, and clearly defined diagnostic criteria. In contrast, many previous studies either focused exclusively on a single condition [3,4,11] or included heterogeneous groups of adults and children [36], thus limiting the interpretability of their findings in different disease contexts.

However, there are several limitations that warrant discussion. The retrospective design limits causal inference and introduces potential selection bias, as children undergoing bronchoscopy generally represent a more severe, persistent, or atypical clinical subset. Given that bronchoscopy with BAL is an invasive procedure, our study population may not capture the full clinical spectrum of PBB or bronchiectasis encountered in general pediatric practice. Consequently, the observed pathogen distribution and prevalence estimates may be influenced by this selection bias, since milder, non-invasively managed cases could display different microbial profiles. Recall bias is also possible due to the retrospective nature of data collection. Moreover, variability in the interval between HRCT and BAL, sometimes extending up to six months, may have influenced concordance between imaging and microbiological findings, as airway infection and inflammation can fluctuate over time. These factors should be considered when interpreting associations between structural findings and microbial results.

While the single-center design ensured procedural consistency, it may limit generalizability to populations with different environmental exposures or pathogen epidemiology. Regional antibiotic prescribing patterns and vaccination coverage in Greece may also have influenced the observed pathogen distribution. Community antibiotic use remains relatively high compared with other European countries, potentially affecting both pathogen prevalence and antimicrobial susceptibility. In addition, widespread implementation of pneumococcal conjugate and *H. influenzae* type b (Hib) vaccination programs has likely reduced carriage and infection by vaccine-covered serotypes, thereby altering the airway microbiological landscape [12]. These regional characteristics should therefore be considered when extrapolating our findings to populations with differing antibiotic stewardship policies or immunization coverage.

Another limitation is that the study relied exclusively on culture-based techniques, which may underestimate the true microbial diversity of the lower airways. Molecular approaches such as 16S rRNA gene sequencing and metagenomics could complement traditional culture by identifying non-culturable bacteria, characterizing microbial community structure, and elucidating host-microbe interactions that sustain chronic infection and inflammation. Recent molecular studies have shown that both PBB and bronchiectasis are characterized by complex polymicrobial biofilm communities dominated by *Haemophilus* species but also containing numerous low-abundance, non-culturable taxa [6,37,38]. These findings suggest that our culture-based approach likely captured the clinically dominant pathogens but underrepresented overall microbial diversity and biofilm complexity. Integrating molecular microbiome profiling with traditional culture in future studies will provide a more comprehensive understanding of airway infection dynamics in pediatric CSLD.

Finally, the absence of data on viral and fungal pathogens represents an additional gap, as these organisms may coexist with bacteria or predispose to secondary infection, potentially underestimating the extent of polymicrobial interactions [39]. Viral and atypical bacterial testing (e.g., *Mycoplasma pneumoniae*, *Chlamydia pneumoniae*, *Bordetella pertussis*) using polymerase chain reaction (PCR) assays was not systematically performed on BAL specimens, reflecting the retrospective design and institutional protocols, in which PCR testing was reserved for acute or severe infections rather than chronic cough evaluation. Consequently, potential viral or atypical bacterial co-detections may have gone undetected. This omission may have underestimated the complexity of polymicrobial interactions within the lower airway microbiome, as viral pathogens can modulate airway immunity, promote bacterial adherence, and contribute to secondary infection. Future studies incorporating multiplex PCR and metagenomic approaches are warranted to elucidate the impact of viral-bacterial co-infections on disease pathophysiology and progression.

In addition, antibiotic susceptibility data were not consistently available across all isolates, precluding assessment of potential differences in antimicrobial resistance between children with PBB and those with bronchiectasis. Detailed information on antibiotic use or hospitalizations during the three months preceding bronchoscopy was also unavailable in some cases, which may have influenced BAL culture yield. Future prospective studies should include systematic susceptibility testing and standardized documentation of recent antibiotic exposure and inpatient care to better characterize resistance patterns and control for potential confounding effects on airway microbiology.

Future research should focus on prospective, multicenter studies incorporating serial BAL sampling, spirometry, molecular microbiology, and clinical outcomes to map the microbiological evolution from PBB to bronchiectasis. Integrating host immune profiling may further clarify why some children with recurrent PBB progress to bronchiectasis, whereas others remain stable. In addition, studies assessing the efficacy of early intervention strategies, such as prolonged antibiotic regimens or adjunctive anti-inflammatory therapies, could identify approaches to halt or even reverse disease progression.

In summary, this study underscores the need for a paradigm shift in the evaluation and management of pediatric chronic wet cough. Clinical phenotype alone should not dictate treatment decisions. Instead, timely and individualized microbiological assessment is essential to optimize outcomes and potentially alter the disease trajectory.

## 5. Conclusions

Our study demonstrates that children with PBB and bronchiectasis share similar lower-airway microbiological profiles, with *H. influenzae* being the predominant pathogen in both conditions. Apart from a higher unadjusted prevalence of *M. catarrhalis* in PBB, no significant differences were observed between groups after adjusting for confounding factors. Younger age was associated with increased odds of polymicrobial infection, suggesting age-related variation in airway microbial ecology.

Overall, these findings support a potential clinical and microbiological continuum between PBB and bronchiectasis but do not establish causality. Importantly, they highlight that early microbiological assessment in children with recurrent or persistent wet cough may help prevent bronchiectasis by enabling timely and targeted antibiotic therapy. Given the substantial overlap in BAL results, the clinical course and chest HRCT findings remain essential for diagnosing bronchiectasis, while BAL continues to provide valuable guidance for antimicrobial selection.

Future research should integrate multi-omic profiling and immunophenotyping to elucidate host-microbe interactions, identify biomarkers predictive of disease progression, and inform personalized treatment strategies for CSLD in children.

## Figures and Tables

**Table 1:** Comparison of demographic and clinical characteristics of the study population.

	PBB (*n* = 85)	BE (*n* = 50)	*p*-Value
Gender			
Male	48 (56.5)	29 (58)	0.862
Female	37 (43.5)	21 (42)	
Age	4.2 (2.2–6.1)	7.8 (5.7–12.1)	**<0.001**
Bhalla score *	-	7 (6–8)	
Comorbidities			
Tracheomalacia/bronchomalacia	35 (41.2)	21 (42)	0.925
Asthma	15 (17.6)	11 (22)	0.536
Lower airway infection **	69 (81.2)	37 (74)	0.327
*H. influenzae*	29 (34.1)	14 (28)	0.461
*S. pneumoniae*	16 (18.8)	4 (8)	0.087
*M. catarrhalis*	18 (21.2)	4 (8)	**0.045**
*H. parainfluenzae*	9 (10.6)	7 (14)	0.554
*P. aeruginosa*	7 (8.2)	7 (14)	0.289
*S. aureus*	11 (12.9)	9 (18)	0.424
Other Gram-negative	7 (8.2)	1 (2)	0.138
Polymicrobial infection ***	22 (25.9)	8 (16)	0.182

Data are presented as *n* (%) and median (IQR); PBB: Protracted bacterial bronchitis; BE: Bronchiectasis; Other Gram-negative: Pathogenic Gram-negative bacteria excluding *H. influenzae*, *H. parainfluenzae*, and *M. catarrhalis* (e.g., Enterobacteriaceae, *Achromobacter xylosoxidans*, *Stenotrophomonas maltophilia*); Numbers in bold indicate *p* < 0.05 * The Bhalla score was applied only to children with bronchiectasis, since by definition structural abnormalities are absent in PBB ** Isolation of any pathogen at ≥10^4^ CFU/mL in BAL fluid *** Isolation of more than one pathogen at significant levels from a single BAL sample.

**Table 2:** Logistic regression analysis of predictors of lower airway and polymicrobial infection.

Outcome	Predictor	OR (95% CI)	*p*-Value
Lower airway infection	Diagnosis (BE vs. PBB)	0.93 (0.35–2.51)	0.888
	Gender (female vs. male)	0.98 (0.42–2.32)	0.971
	Age (years)	0.92 (0.82–1.04)	0.182
	Tracheomalacia/bronchomalacia (yes vs. no)	0.53 (0.23–1.26)	0.152
	Asthma (yes vs. no)	0.84 (0.30–2.31)	0.734
Polymicrobial infection	Diagnosis (BE vs. PBB)	1.15 (0.40–3.32)	0.803
	Gender (female vs. male)	0.96 (0.40–2.26)	0.916
	Age (years)	**0.81 (0.69–0.95)**	**0.009**
	Tracheomalacia/bronchomalacia (yes vs. no)	0.57 (0.23–1.45)	0.240
	Asthma (yes vs. no)	1.61 (0.53–4.92)	0.401

OR: Odds Ratio; 95% CI: 95% Confidence Interval; BE: Bronchiectasis; PBB: Protracted bacterial bronchitis; Lower airway infection is defined as the isolation of any pathogen at ≥10^4^ CFU/mL in BAL fluid; Polymicrobial infection is defined as the isolation of more than one pathogen at significant levels from a single BAL sample; Numbers in bold indicate *p* < 0.05. Model diagnostics indicated adequate fit (Hosmer–Lemeshow *p* > 0.05 for all models) with no multicollinearity (VIFs < 2). Model fit indices demonstrated acceptable explanatory power, with Nagelkerke pseudo-R^2^ = 0.06 for the lower airway infection model and 0.14 for the polymicrobial infection model.

**Table 3:** Adjusted odds ratios (OR) for the detection of specific pathogens in children with bronchiectasis versus protracted bacterial bronchitis.

	OR	95% CI	*p*-Value
*H. influenzae*	1.07	0.43–2.69	0.887
*S. pneumoniae*	0.49	0.14–1.79	0.273
*M. catarrhalis*	1.30	0.33–5.21	0.706
*H. parainfluenzae*	1.55	0.47–5.16	0.474
*P. aeruginosa*	1.06	0.33–3.44	0.917
*S. aureus*	1.32	0.45–3.83	0.613
Other Gram-negative	0.41	0.04–4.19	0.449

OR: Odds Ratio; 95% CI: 95% Confidence Interval; Other Gram-negative: Pathogenic Gram-negative bacteria excluding *H. influenzae*, *H. parainfluenzae*, and *M. catarrhalis* (e.g., Enterobacteriaceae, *Achromobacter xylosoxidans*, *Stenotrophomonas maltophilia*); Model diagnostics indicated adequate fit (Hosmer–Lemeshow *p* > 0.05 for all models) with no multicollinearity (VIFs < 2). Model fit indices demonstrated acceptable explanatory power, with Nagelkerke pseudo-R^2^ values ranging from 0.02 to 0.3 across pathogen-specific models.

## Data Availability

The data presented in this study are available on request from the corresponding author. The data are not publicly available due to privacy and associated regulatory constraints. All patient information was de-identified before data extraction and analysis, and no identifiable personal data was retained. The study fully complied with institutional and national data protection regulations.

## Abbreviations

The following abbreviations are used in this manuscript:

PBB	Protracted bacterial bronchitis
BAL	Bronchoalveolar lavage
HRCT	High-resolution computed tomography
BAR	Broncho-arterial ratio
CSLD	Chronic suppurative lung disease
PCD	Primary ciliary dyskinesia
CF	Cystic fibrosis
ERS	European Respiratory Society
LMA	Laryngeal mask airway
CFU	Colony-forming units
IQR/IQRs	Interquartile ranges
VIFs	Variance inflation factors
OR/ORs	Odds ratios
CI/CIs	Confidence intervals
PCR	Polymerase chain reaction

## References

[1] Chang A.B., Upham J.W., Masters I.B., Redding G.R., Gibson P.G., Marchant J.M., Grimwood K. (2016). Protracted bacterial bronchitis: The last decade and the road ahead. Pediatr. Pulmonol..

[2] Kapur N., Masel J.P., Watson D., Masters I.B., Chang A.B. (2011). Bronchoarterial ratio on high-resolution CT scan of the chest in children without pulmonary pathology: Need to redefine bronchial dilatation. Chest.

[3] Wurzel D.F., Marchant J.M., Yerkovich S.T., Upham J.W., Petsky H.L., Smith-Vaughan H., Masters B., Buntain H., Chang A.B. (2016). Protracted Bacterial Bronchitis in Children: Natural History and Risk Factors for Bronchiectasis. Chest.

[4] Ruffles T.J.C., Marchant J.M., Masters I.B., Yerkovich S.T., Wurzel D.F., Gibson P.G., Busch G., Baines K.J., Simpson J.L., Smith-Vaughan H.C. (2021). Outcomes of protracted bacterial bronchitis in children: A 5-year prospective cohort study. Respirology.

[5] Cole P.J. (1986). Inflammation: A two-edged sword--the model of bronchiectasis. Eur. J. Respir. Dis. Suppl..

[6] Marsh R.L., Binks M.J., Smith-Vaughan H.C., Janka M., Clark S., Richmond P., Chang A.B., Thornton R.B. (2022). Prevalence and subtyping of biofilms present in bronchoalveolar lavage from children with protracted bacterial bronchitis or non-cystic fibrosis bronchiectasis: A cross-sectional study. Lancet Microbe.

[7] Principi N., Esposito S. (2024). Biofilm Production and Its Implications in Pediatrics. Microorganisms.

[8] Pizzutto S.J., Grimwood K., Bauert P., Schutz K.L., Yerkovich S.T., Upham J.W., Chang A.B. (2013). Bronchoscopy contributes to the clinical management of indigenous children newly diagnosed with bronchiectasis. Pediatr. Pulmonol..

[9] Kantar A., Chang A.B., Shields M.D., Marchant J.M., Grimwood K., Grigg J., Priftis K.N., Cutrera R., Midulla F., Brand P.L.P. (2017). ERS statement on protracted bacterial bronchitis in children. Eur. Respir. J..

[10] Pritchard M.G., Lenney W., Gilchrist F.J. (2015). Outcomes in children with protracted bacterial bronchitis confirmed by bronchoscopy. Arch. Dis. Child..

[11] Kapur N., Grimwood K., Masters I.B., Morris P.S., Chang A.B. (2012). Lower airway microbiology and cellularity in children with newly diagnosed non-CF bronchiectasis. Pediatr. Pulmonol..

[12] Priftis K.N., Litt D., Manglani S., Anthracopoulos M.B., Thickett K., Tzanakaki G., Fenton P., Syrogiannopoulos G.A., Vogiatzi A., Douros K. (2013). Bacterial bronchitis caused by Streptococcus pneumoniae and nontypable Haemophilus influenzae in children: The impact of vaccination. Chest.

[13] de Vries J.J.V., Chang A.B., Marchant J.M. (2018). Comparison of bronchoscopy and bronchoalveolar lavage findings in three types of suppurative lung disease. Pediatr. Pulmonol..

[14] de Blic J., Midulla F., Barbato A., Clement A., Dab I., Eber E., Green C., Grigg J., Kotecha S., Kurland G. (2000). Bronchoalveolar lavage in children. ERS Task Force on bronchoalveolar lavage in children. European Respiratory Society. Eur. Respir. J..

[15] Faro A., Wood R.E., Schechter M.S., Leong A.B., Wittkugel E., Abode K., Chmiel J.F., Daines C., Davis S., Eber E. (2015). Official American Thoracic Society technical standards: Flexible airway endoscopy in children. Am. J. Respir. Crit. Care Med..

[16] Radhakrishnan D., Yamashita C., Gillio-Meina C., Fraser D.D. (2014). Translational research in pediatrics III: Bronchoalveolar lavage. Pediatrics.

[17] Gaillard E.A., Kuehni C.E., Turner S., Goutaki M., Holden K.A., de Jong C.C.M., Lex C., Lo D.K.H., Lucas J.S., Midulla F. (2021). European Respiratory Society clinical practice guidelines for the diagnosis of asthma in children aged 5-16 years. Eur. Respir. J..

[18] Makrinioti H., Fainardi V., Bonnelykke K., Custovic A., Cicutto L., Coleman C., Eiwegger T., Kuehni C., Moeller A., Pedersen E. (2024). European Respiratory Society statement on preschool wheezing disorders: Updated definitions, knowledge gaps and proposed future research directions. Eur. Respir. J..

[19] Wallis C., Alexopoulou E., Antón-Pacheco J.L., Bhatt J.M., Bush A., Chang A.B., Charatsi A.M., Coleman C., Depiazzi J., Douros K. (2019). ERS statement on tracheomalacia and bronchomalacia in children. Eur. Respir. J..

[20] Bhalla M., Turcios N., Aponte V., Jenkins M., Leitman B.S., McCauley D.I., Naidich D.P. (1991). Cystic fibrosis: Scoring system with thin-section CT. Radiology.

[21] Pereira F.F., Ibiapina Cda C., Alvim C.G., Camargos P.A., Figueiredo R., Pedrosa J.F. (2014). Correlation between Bhalla score and spirometry in children and adolescents with cystic fibrosis. Rev. Assoc. Med. Bras..

[22] Wiltingh H., Marchant J.M., Goyal V. (2024). Cough in Protracted Bacterial Bronchitis and Bronchiectasis. J. Clin. Med..

[23] Marrella V., Nicchiotti F., Cassani B. (2024). Microbiota and Immunity during Respiratory Infections: Lung and Gut Affair. Int. J. Mol. Sci..

[24] Sánchez Montalvo A., Gohy S., Rombaux P., Pilette C., Hox V. (2022). The Role of IgA in Chronic Upper Airway Disease: Friend or Foe?. Front. Allergy.

[25] Saliu F., Rizzo G., Bragonzi A., Cariani L., Cirillo D.M., Colombo C., Daccò V., Girelli D., Rizzetto S., Sipione B. (2021). Chronic infection by nontypeable Haemophilus influenzae fuels airway inflammation. ERJ Open Res..

[26] Jurcisek J.A., Bakaletz L.O. (2007). Biofilms formed by nontypeable Haemophilus influenzae in vivo contain both double-stranded DNA and type IV pilin protein. J. Bacteriol..

[27] Chang A.B., Fortescue R., Grimwood K., Alexopoulou E., Bell L., Boyd J., Bush A., Chalmers J.D., Hill A.T., Karadag B. (2021). European Respiratory Society guidelines for the management of children and adolescents with bronchiectasis. Eur. Respir. J..

[28] Chang A.B., Oppenheimer J.J., Irwin R.S. (2020). Managing Chronic Cough as a Symptom in Children and Management Algorithms: CHEST Guideline and Expert Panel Report. Chest.

[29] Goyal V., Grimwood K., Marchant J., Masters I.B., Chang A.B. (2014). Does failed chronic wet cough response to antibiotics predict bronchiectasis?. Arch. Dis. Child..

[30] Zemanick E.T., Wagner B.D., Robertson C.E., Ahrens R.C., Chmiel J.F., Clancy J.P., Gibson R.L., Harris W.T., Kurland G., Laguna T.A. (2017). Airway microbiota across age and disease spectrum in cystic fibrosis. Eur. Respir. J..

[31] Atto B., Anteneh Y., Bialasiewicz S., Binks M.J., Hashemi M., Hill J., Thornton R.B., Westaway J., Marsh R.L. (2023). The Respiratory Microbiome in Paediatric Chronic Wet Cough: What Is Known and Future Directions. J. Clin. Med..

[32] Brandtzaeg P. (2013). Secretory IgA: Designed for Anti-Microbial Defense. Front. Immunol..

[33] Simon A.K., Hollander G.A., McMichael A. (2015). Evolution of the immune system in humans from infancy to old age. Proc. Biol. Sci..

[34] Bosch A.A., Biesbroek G., Trzcinski K., Sanders E.A., Bogaert D. (2013). Viral and bacterial interactions in the upper respiratory tract. PLoS Pathog..

[35] Man W.H., de Steenhuijsen Piters W.A., Bogaert D. (2017). The microbiota of the respiratory tract: Gatekeeper to respiratory health. Nat. Rev. Microbiol..

[36] Miao X.Y., Ji X.B., Lu H.W., Yang J.W., Xu J.F. (2015). Distribution of Major Pathogens from Sputum and Bronchoalveolar Lavage Fluid in Patients with Noncystic Fibrosis Bronchiectasis: A Systematic Review. Chin. Med. J..

[37] Tunney M.M., Einarsson G.G., Wei L., Drain M., Klem E.R., Cardwell C., Ennis M., Boucher R.C., Wolfgang M.C., Elborn J.S. (2013). Lung microbiota and bacterial abundance in patients with bronchiectasis when clinically stable and during exacerbation. Am. J. Respir. Crit. Care Med..

[38] Rogers G.B., Carroll M.P., Serisier D.J., Hockey P.M., Jones G., Bruce K.D. (2004). characterization of bacterial community diversity in cystic fibrosis lung infections by use of 16s ribosomal DNA terminal restriction fragment length polymorphism profiling. J. Clin. Microbiol..

[39] Baker C., Chalmers J.D. (2025). Viruses in bronchiectasis. ERJ Open Res..

